# High Burden of Carotid Atherosclerosis in Rural Northeast China: A Population-Based Study

**DOI:** 10.3389/fneur.2021.597992

**Published:** 2021-02-15

**Authors:** Liying Xing, Ru Li, Suli Zhang, Dan Li, Baojing Dong, Hong Zhou, Li Jing, Yuanmeng Tian, Shuang Liu

**Affiliations:** ^1^Department of Cardiology, The First Hospital of China Medical University, Shenyang, China; ^2^Department of Chronic Disease, Liaoning Provincial Center for Disease Control and Prevention, Shenyang, China; ^3^Department of Cardiovascular Ultrasound, The First Hospital of China Medical University, Shenyang, China; ^4^Department of Cardiovascular Ultrasound, Central Hospital of Chao Yang City, Chaoyang, China

**Keywords:** atherosclerosis, epidemiology, carotid arteries, risk factors, China

## Abstract

**Objective:** Carotid atherosclerosis is a known marker of increased cardiovascular risk. We aimed to assess the current epidemiology of carotid atherosclerosis, carotid plaque and related risk factors in rural northeast China.

**Methods:** The population-based, cross-sectional study was conducted in 5,838 adults aged ≥40 years residing in rural northeast China in 2017–2018. A multi-stage cluster sampling method was used to select the representative sample. Carotid atherosclerosis was defined as carotid intima-media thickness (CIMT) ≥1.0 mm or presence of plaque.

**Results:** The mean CIMT was 0.72 ± 0.13 mm and increased with age in this population. Among 2,457 individuals with carotid atherosclerosis, 2,333 were diagnosed with carotid plaque, and 210 individuals were moderate or severe carotid stenosis. Crude prevalence of carotid atherosclerosis and plaque were 42.1 and 40.0%, significantly higher in men than in women (*p* < 0.001). The age-standardized prevalence of carotid atherosclerosis and carotid plaque were 33.1 and 31.5%, respectively. Advancing age, men, hypertension, diabetes, current smoking, ever-smoking and lack of exercise were risk factors for carotid atherosclerosis. Hypertension (69.1%), dyslipidemia (26.0%) and diabetes (16.1%) were highly prevalent in participants with carotid atherosclerosis. However, the control rates of those comorbidities were frustratingly low (4.7, 8.2, and 14.2%, respectively).

**Conclusions:** The high prevalence of carotid atherosclerosis, carotid plaque, carotid stenosis and uncontrolled risk factors indicated the high burden of cardiovascular disease in rural northeast China, particularly in men. Strategies of prevention and management of atherosclerosis and related risk factors were urgently needed in rural northeast China.

## Introduction

Individuals with atherosclerosis have an increasing risk of future cardiovascular morbidity and mortality (1). Many patients with atherosclerosis remain unaware, even those with severe atherosclerosis (2). Therefore, early prevention and management of atherosclerosis might be of great importance in terms of reducing cardiovascular risk.

As a systemic inflammatory vascular disorder, the process of atherosclerosis usually affects arterial beds simultaneously (3). Carotid ultrasound has been proposed as a quick, safe and cost-effective imaging tool for assessing carotid artery atherosclerosis, which is likely to provide the highest yield to reflect the systemic atherosclerosis (2). The ultrasound markers of atherosclerosis include increasing carotid intima-media thickness (CIMT) and plaques (4). An increasing number of studies reported that presence of carotid plaque may be a more powerful prognostic tool of vascular events than IMT (5, 6). Moreover, carotid plaques were significantly associated with cardiovascular disease, and individuals with carotid plaques had a 2.8-fold increase of vascular events in comparison with individuals without carotid plaque (7). Therefore, appropriate population screening for carotid atherosclerosis including plaque could potentially prevent many additional cardiovascular adverse events.

The burden of cardiovascular diseases including atherosclerosis has increased substantially over the past decade in China. However, the information from northeast rural China is still lacking. Previous study indicates rural China has been experiencing rapid epidemiologic transitions and economic progress (8). Majority of the population settle in rural areas in China, and they tend to have low income, low educational levels and poor medical insurance. Therefore, accurate estimation of the current epidemiology of atherosclerosis is critically important for measuring the societal burden and developing evidence-based strategies to reduce the cardiovascular morbidity and mortality in rural northeast China. In the present study, we conducted a cross-sectional survey of a large representative rural northeast China to profile the current prevalence of carotid atherosclerosis and plaque, as well as related risk factors for formulating population-level prevention and management strategies in those areas.

## Methods

### Study Population

**Figure 1:**
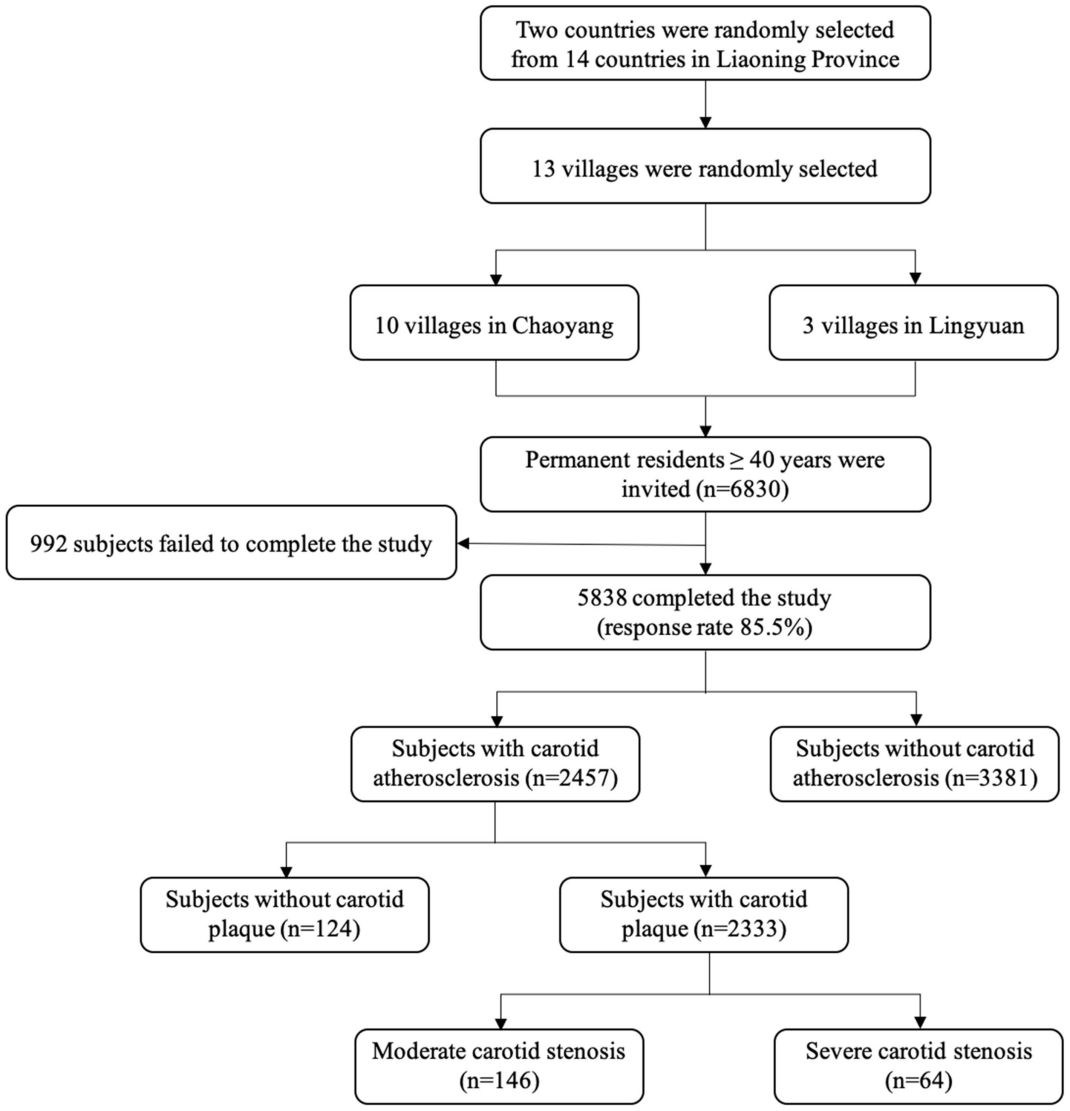
Flow chart of population selection.

This present study derived from a cross-sectional survey conducted in rural areas of northeast China from September 2017 to May 2018. A multi-stage, geologically stratified and cluster random sampling method was employed to ensure the samples were representative. A total of 13 villages were randomly selected from 2 counties (Chaoyang and Lingyuan) of Liaoning province. All permanent residents (individuals registered in the Local Household Register System and living in the selected communities for ≥6 months when the study began) aged more than 40 years old (*n* = 6,830) were recognized as eligible participants. After excluding those individuals who were unwillingness to participate, missing results including blood tests or carotid ultrasound examination, or participants with pregnancy, cancer or mental disorders were excluded, we finally enrolled 5,838 (response rate: 85.5%) individuals into analyses (Figure 1).

### Patient and Public Involvement

The data sets used and analyzed during this study are available from the corresponding author on reasonable request. No patients were directly involved in setting the research question, developing plans for recruitment, design or implementation of this study. No patients were asked to advise on interpretation or writing of the results. There are no specific plans to disseminate the results of the research to study participants.

### Definitions

Participants were asked whether they regularly consumed alcohol and cigarettes. Current smoking (≥1 cigarette/day for at least 6 months) and current drinking (any dose of alcohol, ≥1 time/week) were defined according to the participants' self-report (9). Ever smoking was defined when participants had a history of smoking but stopped smoking for at least 6 months previous to the survey. Regular exercise was identified as moderate intensity exercise (equivalent to walking) for ≥30 min and ≥3 times per week, which moderate and heavy manual workers fulfill during their work (10). Lack of exercise was defined as failing to meet the above standards for regular exercise. Overweight or obese was defined as a BMI ≥ 24 kg/m^2^ according to the criteria recommended by Working Group on Obesity in China (11).

Hypertension was defined as a mean systolic blood pressure (SBP) ≥140 mmHg or a mean diastolic blood pressure (DBP) ≥90 mmHg, and/or self-reported use of antihypertensive medication in the past 2 weeks (12). Treatment of hypertension was defined as use of antihypertensive drugs in the past 2 weeks. Hypertension control was defined as an average SBP < 140 mmHg, and an average DBP < 90 mmHg.

Dyslipidemia was diagnosed if the participants met one of the following criteria: (1) High TC: TC ≥ 6.22 mmol/L (240 mg/dL); (2) high TG: TG ≥ 2.26 mmol/L (200 mg/dL); (3) high LDL-C: LDL-C ≥ 4.14 mmol/L (160 mg/dL); (4) low HDL-C: LDL-C < 1.04 mmol/L (40 mg/dL); and (5) self-reported use of lipid-regulating medications (13). Treatment of dyslipidemia was defined as use of lipid regulating drugs in the past 12 weeks. Dyslipidemia control was defined as the participant's serum lipid levels do not meet the criteria of dyslipidemia anymore, otherwise it was considered as uncontrolled.

Diabetes mellitus (DM) was diagnosed as an FBG ≥7.0 mmol/L or HbA1c ≥6.5%, and/or self-reported diagnosis that was previously determined by a physician (14). Treatment of DM was defined as receiving diabetes medications. DM control was defined as DM patients with HbA1c <7.0%. Patients who did not meet this criterion were considered as uncontrolled DM. The detailed methods of data collection have been described previously (15).

### Ultrasound Protocol

All scans were performed using a high-resolution B-mode ultrasonography (Mindray M7, Shenzhen, China) with a broadband 7L4S linear array transducer. Carotid ultrasound was performed by certified sonographers with >3 years of experience in vascular ultrasound imaging, all the sonographers underwent standard training before data collection, and completed pilot examination (performed with volunteers). Further instruction and support were provided by study authors during data collection. All subjects were examined in prone position with both left and right carotids scanned. A transverse scan starting at the clavicle and moving cranially up to the mandible was performed for orientation firstly, thereby longitudinal images of carotid artery as well as plaque was obtained subsequently.

Plaque was displayed both in short-axis view and long-axis sweep. Color and pulsed Doppler imaging were obtained if necessary. The highest peak systolic and end-diastolic velocity measurements from the common carotid artery and the internal carotid artery were obtained to confirm the degree of carotid stenosis (16). The Doppler velocity thresholds was used to determine the degree of stenosis according to the criteria that were previously described. Moderate carotid stenosis was defined as the degree of carotid artery stenosis between 50 and 69%, and severe carotid stenosis was defined as the degree of carotid stenosis ≥70% (16, 17).

Carotid atherosclerosis was defined as mean CIMT ≥ 1.0 mm and/or presence of carotid plaque (16, 18). For each participant, CIMT was measured within a region free of plaque from a common carotid artery video acquisition according to Mannheim consensus (18). Three mean CIMT measurements were taken on each artery (left/right side), and CIMT measurements from both sides were averaged to create mean CIMT. The presence of plaques was assessed in the common carotid artery, bulb, and internal carotid artery segments, and defined as a focal thickness of CIMT ≥ 1.5 mm, or focal intraluminal protrusion >50% of the surrounding CIMT according to Mannheim carotid intima-media thickness and plaque consensus (18).

### Statistical Analysis

Descriptive statistics were calculated for all variables. Continuous variables with normal distribution are reported as means and standard deviations, other continuous variables are reported as medians and interquartile ranges if appropriate. Categorical variates were summarized as frequencies and percentages. Differences between groups were compared using a χ^2^ test for categorical variables. Univariate and stepwise multivariate logistic regression analyses were performed to evaluate the association between risk factors and carotid atherosclerosis. The intra-reader variability of the measurements was determined for the CIMT in the CCA of 50 individuals after this study. CIMT was examined twice after a 1-week interval in a blinded manner. Variability was determined by calculating coefficients of variation, which was calculated as the standard deviation of differences. Paired *t*-test was employed to test the difference between the two measurements. All statistical analyses were conducted by using SPSS22.0 (SPSS Inc., Chicago, IL, USA); *P*-values < 0.05 were considered statistically significant.

## Results

### Characteristics of the Population

The characteristics of the study was shown in Table 1. The 5,838 participants included 2,296 men (39.3%) and 3,542 women (60.7%). The average age of the whole population was 59.0 ± 10.1 years. In our study population, 57.6% had a primary school education or less, and 36.5% had an annual income <5,000 yuan (~$700).

**Table 1:** Characteristics of the study participants.

	**With carotid atherosclerosis**	**Without carotid atherosclerosis**	**Total**	***P*-value**
Participant, *n* (%)	2,457 (42.1)	3,381 (57.9)	5,838 (100.0)	
**Sex**				
Men	1,084 (32.1)	1,212 (49.3)	2,296 (39.3)	<0.001
Women	2,297 (67.9)	1,245 (50.7)	3,542 (60.7)	
**Mean age, years**	64.8 ± 9.0	54.7 ± 8.7	59.0 ± 10.1	<0.001
40–49	118 (4.8)	1,062 (31.4)	1,180 (20.2)	<0.001
50–59	519 (21.1)	1,305 (38.6)	1,824 (31.2)	
60–69	1,077 (43.8)	836 (24.7)	1,913 (32.8)	
70–79	609 (24.8)	156 (4.6)	765 (13.1)	
≥80	134 (5.5)	22 (0.7)	156 (2.7)	
**Education**, ***n*** **(%)**				
Primary school or lower	1,632 (66.4)	1,730 (51.2)	3,362 (57.6)	<0.001
Middle school	787 (32.0)	1,472 (43.5)	2,259 (38.7)	
High school or above	38 (1.5)	179 (5.3)	217 (3.7)	
**Incomings per year, Yuan**				
<5,000	1,119 (45.5)	1,012 (29.9)	2,131 (36.5)	<0.001
5,000–9,999	590 (24.0)	768 (22.7)	1,358 (23.3)	
10,000–19,999	420 (17.1)	752 (22.2)	1,172 (20.1)	
≥20,000	328 (13.3)	849 (25.1)	1,177 (20.2)	
BMI, kg/m^2^	24.1 ± 3.6	25.1 ± 3.8	24.7 ± 3.7	<0.001
<18.5	119 (4.8)	77 (2.3)	196 (3.4)	<0.001
18.5–23.9	1,127 (45.9)	1,286 (38.0)	2,413 (41.3)	
24.0–27.9	880 (35.8)	1,292 (38.2)	2,172 (37.2)	
≥28.0	331 (13.5)	726 (21.5)	1,057 (18.1)	
Mean SBP, mmHg	148.4 ± 22.7	136.7 ± 21.5	141.7 ± 22.8	<0.001
Mean DBP, mmHg	88.2 ± 11.8	86.6 ± 11.6	87.3 ± 11.8	<0.001
Current smoking, *n* (%)	777 (31.6)	724 (21.4)	1,501 (25.7)	<0.001
Current drinking, *n* (%)	743 (30.2)	973 (28.8)	1,716 (29.4)	0.226
Lack of Exercise, *n* (%)	504 (20.5)	352 (10.4)	856 (14.7)	<0.001
Hypertension, *n* (%)	1,697 (69.1)	1,661 (49.1)	3,358 (57.5)	<0.001
Diabetes, *n* (%)	393 (16.1)	350 (10.4)	743 (12.8)	<0.001
High TC, *n* (%)	333 (13.6)	360 (10.7)	693 (11.9)	0.001
High TG, *n* (%)	333 (13.6)	495 (14.7)	828 (14.2)	0.244
Low HDL, *n* (%)	18 (0.7)	27 (0.8)	45 (0.8)	0.778
High LDL, *n* (%)	56 (2.3)	66 (2.0)	122 (2.1)	0.386

### Prevalence of Carotid Atherosclerosis and Carotid Plaque

Overall, the mean CIMT value was 0.72 ± 0.13 mm. The correlation coefficient between the first and second CIMT measurements was 0.88 (*p* > 0.05), with no significant difference. The mean difference and standard deviation was 0.02 ± 0.06 mm.

A total of 2,457 were diagnosed with carotid atherosclerosis, and 2,333 participants had carotid plaque as shown in Table 2. The overall crude prevalence of carotid atherosclerosis and plaque were 42.1% (95%CI: 40.8–43.4%) and 40.0% (95%CI: 38.1–41.2%), respectively, significantly higher in men than in women (52.8 vs. 25.1% and 50.0 vs. 33.5%, *p* < 0.001, respectively). The prevalence of carotid atherosclerosis and carotid plaque rose steeply with advancing age (*p* < 0.001 respectively), from 9.4% (95% CI, 7.7–11.1%) among those 40–49 years old to 83.3% (95% CI, 80.4–91.4%) among those ≥ 80 years.

**Table 2:** Prevalence of carotid atherosclerosis and carotid plaque among adults living in rural areas of Northeast China by sex in 2017–2018.

**Age group (years)**	**Men**		**Women**		**Total**		
	**Rate**	**95%CI**	**Rate**	**95%CI**	**Rate**	**95%CI**	***P*-value**
**Carotid atherosclerosis**							
40–49	17.9	14.0–21.9	6.4	4.7–8.1	10.0	8.3–11.7	<0.001
50–59	38.0	34.4–41.7	22.8	20.4–25.3	28.5	26.4–30.5	<0.001
60–69	64.7	61.4–67.9	49.7	46.7–52.7	56.3	54.1–58.5	<0.001
70–79	82.5	78.5–86.5	77.2	73.2–81.3	79.6	76.7–82.5	0.073
≥80	89.4	81.8–97.0	83.3	75.5–91.2	85.9	80.4–91.4	0.283
**Total**	52.8	50.7–54.8	35.1	3.6–36.7	42.1	40.8–43.4	<0.001
ASR*	40.4	38.4–42.4	29.2	27.7–30.7	33.1	32.4–34.8	
**Carotid plaque**							
40–49	16.8	13.0–20.7	6.0	4.4–7.7	9.4	7.7–11.1	<0.001
50–59	36.0	32.3–39.6	21.8	19.4–24.2	27.0	25.0–29.1	<0.001
60–69	61.2	57.9–64.5	46.6	43.7–49.6	53.1	50.8–55.3	<0.001
70–79	78.4	74.1–82.8	74.6	70.4–78.8	76.3	73.3–79.4	0.210
≥80	83.3	74.1–92.6	83.3	75.5–91.2	83.3	77.4–89.2	1.000
**Total**	50.0	47.9–52.0	33.5	31.9–35.0	40.0	38.7–41.2	<0.001
ASR*	38.1	36.1–40.1	27.9	26.4–29.4	31.5	30.3–32.7	

ASR^*^, Age standardized rates by China census population 2010.

The age-standardized prevalence of carotid atherosclerosis and that of carotid plaque were 33.1% (95%CI: 32.4–34.8%) and 31.5% (95%CI: 30.3–32.7%), respectively. The prevalence of carotid atherosclerosis for men and women were 40.0% (95%CI: 38.4–42.4%) and 29.2 (95% CI: 27.7–30.7%), respectively, while the prevalence of carotid plaque for men and women were 38.1% (95%CI: 36.1–40.1%) and 27.9% (95%CI: 26.4–29.4%), respectively.

**Figure 2:**
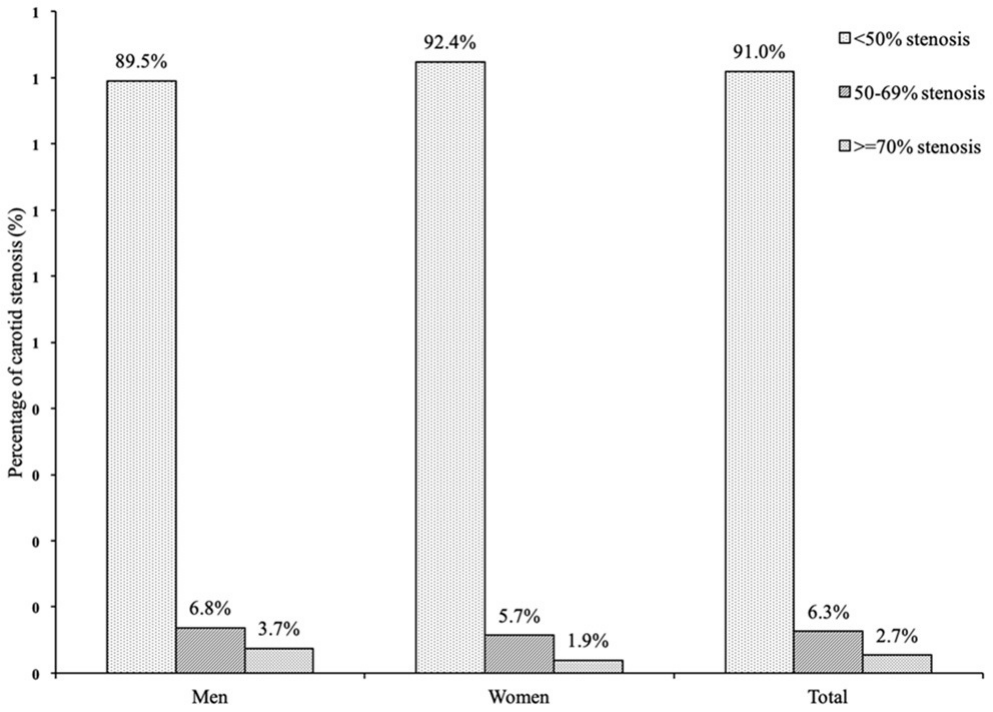
The distribution of carotid stenosis in the population with carotid plaque in rural northeast China.

Among participants with carotid plaque, 210 were diagnosed with moderate or severe carotid stenosis. The percentage of moderate or severe carotid stenosis was estimated to be 9.0%. The percentages of moderate- and severe- carotid stenosis were significantly higher in men than in women (10.5 vs. 7.4%, *p* = 0.01) (Figure 2).

### Related Risk Factors for Prevalence of Carotid Atherosclerosis

According to the results of the multivariate regression model (Table 3), we identified that advancing age (OR, 3.11, 95% CI: 2.49–3.88; OR, 9.05, 95% CI: 7.24–11.31; OR, 26.03, 95% CI: 19.74–34.33; OR, 38.20, 95% CI: 22.93–63.65 in those 50–59, 60–69, 70–79, and ≥80 years old groups, respectively, when compared to those 40–49 years old group), men (OR, 1.72; 95% CI, 1.42–2.06), hypertension (OR, 1.57; 95% CI, 1.38–1.79), diabetes (OR, 1.42; 95% CI, 1.19–1.69), current smoking (OR, 1.63; 95% CI, 1.35–1.96), ever smoking (OR, 1.34; 95% CI, 1.05–1.71) and lack of exercise (OR, 1.22; 95% CI, 1.01–1.46) were risk factors for presence of carotid atherosclerosis. What is noteworthy is that participants with a higher education level (OR, 0.65; 95% CI, 0.43–0.98) were likely to have low prevalence of carotid atherosclerosis in rural northeast China.

**Table 3:** Multivariable-adjusted odds ratio for prevalence of carotid atherosclerosis in rural Northeast China.

	**Men**			**Women**			**Total**		
	**OR**	**95% CI**	***P*-value**	**OR**	**95% CI**	***P*-value**	**OR**	**95% CI**	***P*-value**
**Sex**									
Women	–	–		–	–		1.00	–	
Men	–	–		–	–		1.72	1.43–2.06	<0.001
**Age group, years**									
40–49	1.00	–		1.00	–		1.00	–	
50–59	2.59	1.89–3.55	<0.001	3.89	2.84–5.33	<0.001	3.11	2.49–3.88	<0.001
60–69	7.45	5.40–10.29	<0.001	12.03	8.82–16.40	<0.001	9.05	7.24–11.31	<0.001
70–79	18.88	12.53–28.44	<0.001	39.25	26.98–57.09	<0.001	26.03	19.74–34.33	<0.001
≥80	36.42	15.64–84.82	<0.001	51.07	26.86–97.11	<0.001	38.20	22.93–63.65	<0.001
**Education**									
Primary school or lower	–	–		–	–		1.00	–	
Middle school	–	–		–	–		0.91	0.79–1.04	0.164
High school or above	–	–		–	–		0.65	0.43–0.98	0.040
**Incoming, Yuan**									
<5,000	1.00	–							
5,000–9,999	1.18	0.92–1.51	0.207	–	–		–	–	
10,000–19,999	1.28	0.98–1.68	0.074	–	–		–	–	
≥20000	0.86	0.65–1.14	0.293	–	–		–	–	
**Lack of exercise**									
No	–	–		1.00	–		1.00	–	
Yes	–	–		1.26	1.00–1.58	0.053	1.22	1.01–1.46	0.036
**Smoking**									
Never smoke	1.00	–		1.00	–		1.00	–	
Current smoking	1.79	1.42–2.26	<0.001	1.50	1.08–2.08	0.015	1.63	1.35–1.96	<0.001
Ever smoked	1.69	1.27–2.25	<0.001	0.68	0.40–1.15	0.149	1.34	1.05–1.71	0.017
**Current drinking**									
No	1.00	–		–	–		1.00	–	
Yes	0.81	0.67–0.98	0.031	–	–		0.85	0.72–0.99	0.041
**Hypertension**									
No	1.00	–		1.00	–		1.00		
Yes	1.54	1.27–1.86	<0.001	1.63	1.38–1.94	<0.001	1.57	1.38–1.79	<0.001
**Diabetes**									
No	1.00	–		1.00	–		1.00	–	
Yes	1.39	1.04–1.85	0.026	1.47	1.18–1.84	0.001	1.42	1.19–1.69	<0.001
**High TC**									
No	–	–		–	–		1.00	–	
Yes	–	–		–	–		1.19	0.99–1.43	0.068

### Prevalence of Risk Factors in Patients With Carotid Atherosclerosis

Among risk factors analyzed, the prevalence of hypertension, diabetes and dyslipidemia among people with carotid atherosclerosis was high, accounting for 69.1, 16.1, and 26.0%, respectively (Table 4). The proportion of current smoking, and obesity or overweight among individuals with carotid atherosclerosis was 31.6, and 49.3%, respectively. Women were more likely to have hypertension, diabetes, dyslipidemia and overweight or obesity than men. However, compared with men, women had a significantly lower prevalence of current smoking.

**Table 4:** Prevalence of main risk factors among participants with carotid atherosclerosis by sex in rural Northeast China.

	**Men**		**Women**		**Total**		***X*^**2**^**	***P*-value**
	***N***	**Rate**	***N***	**Rate**	***N***	**Rate**		
Hypertension	807	66.6	890	71.5	1697	69.1	6.907	0.009
Diabetes	158	13.1	235	18.9	393	16.1	15.378	<0.001
Dyslipidemia	243	20.1	394	31.7	637	26.0	42.577	<0.001
High TC	109	9.0	224	18.0	333	13.6	42.145	<0.001
High TG	126	10.4	207	16.7	333	13.6	20.135	<0.001
Low HDL	10	0.8	8	0.6	18	0.7	0.287	0.592
High LDL	18	1.5	38	3.1	56	2.3	6.722	0.010
Current smoking	683	56.4	94	7.6	777	31.6	676.455	<0.001
Alcohol drinking	641	52.9	102	8.2	743	30.2	581.562	<0.001
Overweight or obesity	503	41.5	708	56.9	1,211	49.3	58.013	<0.001

**Figure 3:**
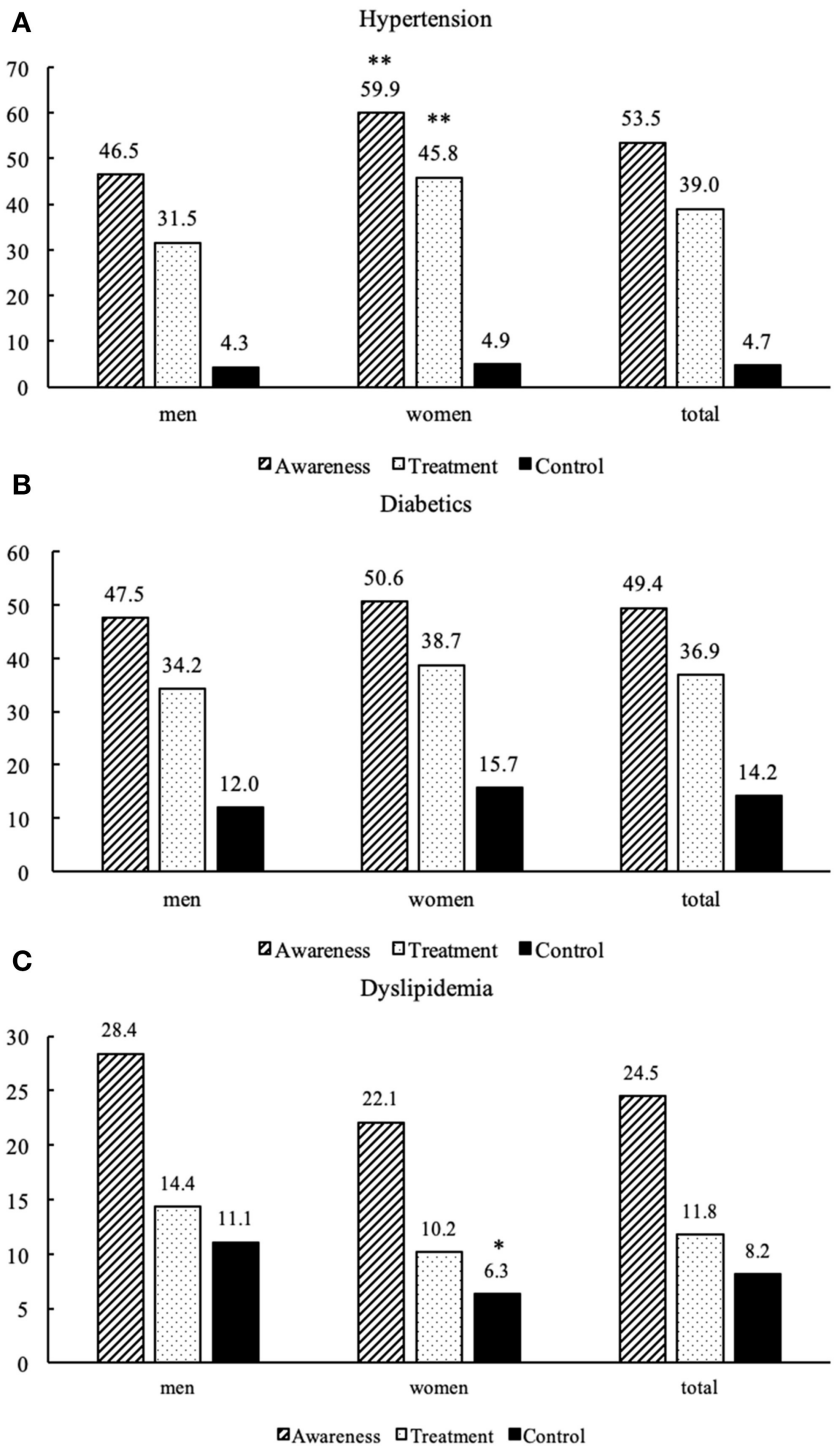
Awareness, treatment, and control of hypertension **(A)**, diabetics **(B)**, and dyslipidemia **(C)** among participants with carotid atherosclerosis in rural Northeast China. **p < 0.001 indicates women vs. men; *p < 0.05 indicates women vs. men.

Overall, 53.3, 49.4, and 24.5% of those with carotid atherosclerosis were aware of their hypertension, diabetes and dyslipidemia specifically. However, only 39.0, 36.9, and 11.8% were treated medically, and 4.7, 14.2, and 8.2% had adequate control. The control rate of dyslipidemia was lower in women (11.1 vs. 6.3%, *p* = 0.033). However, the control rates of hypertension and diabetes did not reach statistical significance between men and women (Figure 3).

## Discussion

The principal findings of the present study are that the high percentages of individual have carotid atherosclerosis and carotid plaque in rural northeast China based on a large contemporary population estimation. The high prevalence of carotid atherosclerosis, carotid plaque, carotid stenosis and related risk factors have become a public health challenge and great economic burden in those areas, especially in men. Therefore, strategies focusing on prevention and detection of atherosclerosis and risk factors, in addition to qualified management of related risk factors, should be emphasized in rural northeast China.

The strength of the present study is that we provided a foundation for the evaluation the current carotid atherosclerosis status in a highly representative population in northeast China. Previous studies on the epidemiology of carotid atherosclerosis were limited by relatively small sample size (19), non-representative sampling (20), restricted region and population (21), or lack of standardized diagnostic criteria (22). On the contrary, the research method and patient population of the study were standard, which allowed a relatively precise estimation of recent epidemiology of carotid atherosclerosis.

Atherosclerosis has been recognized as a chronic inflammatory reaction that involves multiple interactions among immune, vascular and metabolic systems (7, 23). Existence of carotid plaque and/or cIMT thickness has long been considered as validated marker of vascular disease. In contrast to the carotid atherosclerosis prevalence (41.6%) revealed in the survey conducted in the rural China, which was 41.6% (20), the prevalence of carotid atherosclerosis (33.1%) was lower than the national average level, possibly because they excluded participants with stroke or coronary heart disease.

Carotid plaque consists of intimal thickening, foam cells, smooth muscle cells, macrophages, lipid core and fibrous cap, which represents distinctive phenotype of atherosclerosis, have a great likelihood of rapid progression, rupture and thrombotic complications (4, 7). Moreover, carotid plaque is strongly influenced by known risk factors such as hypertension, diabetes and dyslipidemia. In comparison, cIMT is usually identified by genetic determinants and reflects non-atherosclerotic compensatory remodeling rather than early atherosclerosis in many cases (24). In our study, the prevalence of carotid plaque was significantly higher than the national level reported 16.5% in 2018 (20). The China Kadoorie Biobank study (CKB study) previously indicated carotid plaque was present in 31% in multiple geographic regions of China, which roughly consist with the age-specific prevalence in our study. Moreover, compared with carotid atherosclerosis prevalence of 18 and 42% according to the large-scale, cross-sectional study from India and North America (25), the prevalence of carotid plaque in rural northeast China was high. Therefore, the burden of carotid atherosclerosis in rural northeast China remains high, and effective strategies to prevent and manage atherosclerosis should be highlighted.

The Global Burden of Disease study showed that the burden of cardiovascular diseases has increased substantially over the past decade, and the deaths caused by cardiovascular disease increased from 17.7 million in 2008 to 25 million in 2020, suggesting insufficient effective in preventing cardiovascular events (26). Even in subjects in low and moderate cardiovascular risks, more than 50% of them were assigned into the high-risk population according to the presence of carotid atherosclerosis (23). Given the high prevalence of atherosclerosis and carotid plaque in this population and in light of the higher predictive value of plaque for incident cardiovascular events, it may be appropriate to screen for the presence of carotid atherosclerosis by ultrasound in large-scale population to improve risk stratification (4). In our cohort, we also found a high percentage of carotid stenosis among people with carotid plaques, indicating that appropriate population screening for carotid plaque could potentially prevent many additional cardiovascular adverse events and reduce burden of cardiovascular disease in rural China (27).

Our study also identified that age, men, hypertension, diabetes, dyslipidemia, current smoking, ever-smoking and lack of excise were risk factors possibly contributing to high prevalence of carotid atherosclerosis. It is well known that advancing age is the most significant risk factor for atherosclerosis. This might attribute to the longer exposure to various atherosclerotic risk factors and aging-associated functional deterioration (28). Sex-specific prevalence in atherosclerosis has been well described, and women have a relatively lower prevalence of atherosclerosis as well as clinical events than men. The estrogen levels and differences in psychosocial factors and vascular biology might account for this difference (29). Noticeably, our result suggested participants with a higher education level were likely to have low prevalence of carotid atherosclerosis, possibly because of less relationship stress and unhealthy lifestyles in this population. Moreover, according to our previous study, individuals with high education were likely to have low levels of blood pressures, and high control rate of hypertension, which might also contribute to the low prevalence of carotid atherosclerosis (30).

Additionally, we observed high prevalence of risk factors in carotid atherosclerosis populations in the present study, which suggested rural China lagged in risk factor management. The poor management of the risk factors of atherosclerosis was likely to associate with the increased cardiovascular risk in the future. Therefore, adequate screening and treatment of risk factors of atherosclerosis were required to reduce the burden in rural northeast China.

This present study also had several limitations. Firstly, because of the cross-sectional design, we can only provide implications for the association between carotid atherosclerosis and cardiovascular events, but the causality of this association still needs more longitudinal studies to validate. Secondly, selection bias was inevitable in the study. However, we made many efforts to minimize the possibility including cross-checking of the data from door-to-door interviews, involvement of professional clinical physicians and local government. Thirdly, our population originated from the rural areas of northeast China. Therefore, whether our results are suitable to the population in different geographic and economic conditions also needs further studies to confirm.

## Conclusions

The present study provides insight into the presence of carotid atherosclerosis and related risk factors in a large representative population in rural northeast China, thereby allowing appropriate implementation of further cardiovascular events prevention and management strategies in those areas. We observe a relatively high prevalence of carotid atherosclerosis, carotid plaque and carotid stenosis. Moreover, the poor management of risk factors including hypertension, diabetes and dyslipidemia might contribute to the considerable burden of cardiovascular disease in the future in rural areas. Therefore, strategies of prevention and management of atherosclerosis as well as related risk factors were urgently needed in rural northeast China.

## Data Availability Statement

The raw data supporting the conclusions of this article will be made available by the authors, without undue reservation.

## Ethics Statement

The studies involving human participants were reviewed and approved by the China National Center for Cardiovascular Disease. Written informed consent was obtained from all participants. The patients/participants provided their written informed consent to participate in this study.

## Author Contributions

SL and LX were responsible for the concept and design of the study and contributed to the drafting of the manuscript. LX was responsible for the study coordination and conduct. RL, SZ, DL, and BD collected and analyzed the data. SL, LX, LJ, and YT interpreted the data. All authors contributed to the article and approved the submitted version.

## Conflict of Interest

The authors declare that the research was conducted in the absence of any commercial or financial relationships that could be construed as a potential conflict of interest.
